# Research on the impact of the urban park built environment on physical activity

**DOI:** 10.1038/s41598-025-13724-7

**Published:** 2025-08-05

**Authors:** Zhixia Fan, Ying Lv, Li Guo

**Affiliations:** College of Landscape Architecture, Sichuan Agricultural University, Chengdu, 611130 China

**Keywords:** Urban park, Built environment, Physical activity, Environmental social sciences, Health care

## Abstract

Urban parks provide important places for the public to engage in physical activities. However, certain aspects of the built environment may affect residents’ physical activities in these parks, and these aspects may vary depending on the city context. Studies have not yet fully explored the factors affecting physical activities in urban parks in Chengdu, China. Furthermore, an in-depth analysis and exploration of the causes behind how the park environment affects physical activity is lacking. This study focused on five urban parks in Chengdu City. Specifically, logistic regression was applied to questionnaire data to study how the built environment of the urban parks affects the duration and frequency of residents’ physical activities. The results indicated that the built environment of the five parks has a significant effect on both duration and frequency. Specifically, the primary factors affecting the duration of physical activities include sanitation maintenance, plant landscape, the presence of a playground, site facilities, lighting facilities, and public toilet facilities. The frequency of physical activities is significantly impacted by pathway maintenance, the playground, pathway width, pathway length, site facilities, and water quality management. The study also explored the causes of how the built environment affects physical activity, proposing several design strategies. These strategies include improving the quantity and quality of the park site and facilities, designing suitable roads, maintaining the environment, and providing an attractive landscape.

## Introduction

In 2020, the Report of the National Health Council on Nutrition and Chronic Disease of Chinese Residents highlighted obesity as a problem in the Chinese population, with a corresponding increase in the prevalence of and morbidity from chronic diseases. Chronic diseases have become a primary threat to public health; deaths due to chronic diseases accounted for 88.5% of total deaths in China in 2019^1^. The main cause was cited to be insufficient physical activity^2^. Urban parks provide free and accessible green spaces in cities, and studies have identified them as important settings for physical activity^3–5^. Physical activity in parks significantly reduces stress and restores attention^6,7^. The pleasant multi-sensory stimuli, including the air, humidity, and visual aesthetics, increase the effectiveness of physical activity and promote health benefits^8^.

Many studies have explored the relationship between the built environment of an urban park and physical activity. With respect to spatial dimensions, Cervero proposed a classic 5D framework for the built environment: density, diversity, design, distance to transit, and destination accessibility. However, studies have yielded mixed findings concerning the impact of park size, proximity, distance, and density^9–11^. At a site-specific level, play facilities and perceived park safety have been reported to be the primary factors affecting physical activity^3^. Other park features that encourage physical activity include excellent maintenance, a relaxing atmosphere, and shady trees^12^, as well as the availability of playground/outdoor fitness equipment^13^, diverse facilities^14^, walking paths, and nature and vegetation^15^. This study focused on the site level. Based on previous research, the built environment was categorized into the five dimensions of pathway design, park maintenance, park landscape, park facilities, and activity sites.

Many studies have explored the duration and frequency of physical activity. For example, Kacynski used self-reporting to capture the duration and frequency of participant exercise^14^. Kong conducted a questionnaire survey to explore the duration and frequency of residents’ physical activities in urban community parks in Jinan City, China^16^. Duration and frequency have been found to be significantly related to physical activity: participants visiting a park more frequently and for longer report more physical activity ^17^. The frequency and duration of park visits have also been found to be significantly associated with participant life satisfaction^17^. Data for these indicators are relatively easy to obtain; therefore, this paper measured physical activity by focusing on duration and frequency.

Many studies have shown that the factors affecting physical activity vary by city. For example, Aliyas et al. found that a larger size, better facilities, and more pleasing aesthetics increased the odds of public open spaces being more active in Iran^18^. Conversely, a study by Arifwidodo found that adding more facilities in a park in Bangkok, Thailand, did not increase the likelihood of visitors engaging in moderate to vigorous physical activity^11^. In Changchun, China, in addition to play facilities, perceived safety was found to play a significant intermediary role in children’s physical activity^3^. However, Cohen found that perceptions of park safety were not associated with park use in a Southern California metropolitan area park in the United States^19^.

With respect to Chengdu, researchers have considered the peri-urban area, neighborhood and street-level built environment, and physical activity, which includes specific activities such as the collective walking behavior and fitness jogging^20,21^. In terms of the urban park built environment, Li et al. analyzed the relationship between characteristics of the park ecosystem and public behavior and preferences^22^. However, few studies have examined the relationship between the built environment and physical activity in Chengdu urban parks. Chengdu, the capital of China’s Sichuan Province, is a park city containing more than 20 million people and over 1,500 urban parks^23^. Ranked as one of the top new first-tier cities, Chengdu excels in terms of commercial resource concentration, urban hubs, and future plasticity^24^. Thus, studying the built environment of parks that affect residents’ physical activities in Chengdu is very meaningful.

To address the above topics, a comprehensive and systematic study on the effects of the urban park built environment on physical activity in Chengdu City was conducted. The study (1) identifies how built environment factors influence residents’ physical activities in the parks, (2) conducts an in-depth analysis of the causes of how the parks’ built environments influence physical activity, and (3) discusses design optimization strategies for Chengdu city parks. The results provide theoretical and practical support for the planners of urban parks in Chengdu, focusing in particular on increasing the duration and frequency of physical activity in parks to improve resident health. The results also provide a theoretical and practical reference for designing and managing other urban parks.

## Materials and methods

### Overview of study area and park selection

Chengdu, located in western China, has a subtropical monsoon climate and four distinct seasons. The annual average temperature is 15.7–17.7℃; the total precipitation is 798.3–1541.0 mm; and the annual average sunshine duration is 685.5–1002.9 h^25^. As the economic, cultural, and transport center of southwest China, Chengdu has topped the list of new first-tier cities for ten consecutive years^26^. As of the end of 2023, Chengdu had a population of 21.403 million^27^ and a gross domestic product (GDP) ranked seventh in China. A survey reported that more than 70% of middle-aged people in Chengdu engage irregularly in body-building activities, with an even lower participation rate by 40–45-year-olds^28^ .

Chengdu has more than 1,500 urban parks. Five of those parks were examined in this study, including two comprehensive parks, one community park, one specialized park, and one street park. These parks are located in the main urban area, which includes the urban areas and ring city ecological zones of all municipal districts (12 districts of Jinjiang, Qingyang, Jinniu, Wuhou, Chenghua, Longquanyi, Qingbaijiang, Xindu, Wenjiang, Shuangliu, Pixi, and Xinjin, plus the Chengdu High-Tech Zone and Sichuan Tianfu New Area Directly Administered Area), covering an area of about 1564 square kilometers^29^. These parks are free, accessible by public transportation, and surrounded by a diverse residential, educational, and commercial area with high pedestrian flow. Together, they represent the diversity of the city’s urban parks.

**Fig. 1:**
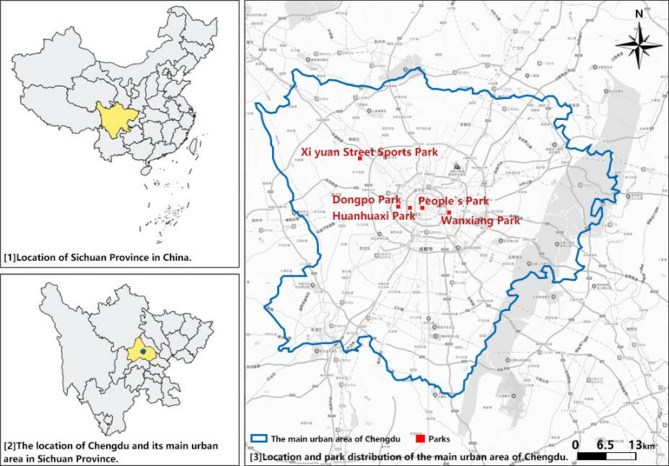
Location of the study area and research sites (Source: Drawn by author).

**Fig. 2:**
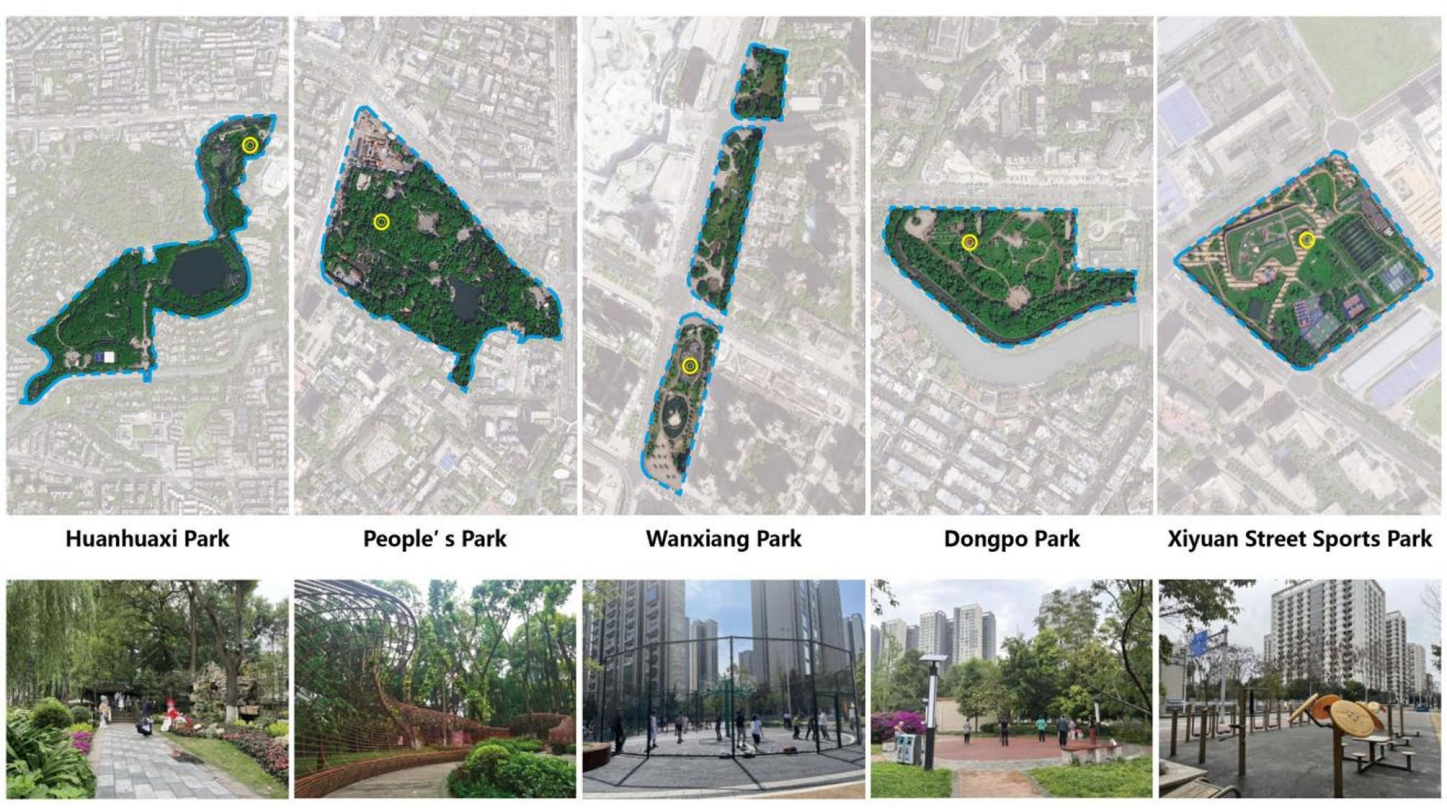
Photos of the five studied urban parks.

The five parks included in this study are the Huanhuaxi Park, People’s Park, Dongpo Park, Xiyuan Street Sports Park, and Wanxiang Park. Huanhuaxi Park and People’s Park are comprehensive parks, popular among locals and foreign tourists. They are both large, with Huanhuaxi Park in particular covering an area of 32.32 hm^2^. Dongpo Park, a community park located between the second and third ring roads in Chengdu, is mostly surrounded by residential and commercial areas. The Xiyuan Street Sports Park is a sports-specific park. Wanxiang Park is a street park in the core area of the Huarun Vientiane City cluster in Chenghua District, next to Shuangcheng Er Road (Fig. 1). Information about these five parks is shown in Fig. 2 and Table 1.

**Table 1:** Research park information.

Park name	Park area (hm^2^)	Park type	Main road width (m)	Green coverage (%)	Site size (m^2^)	Density of public toilet facilities (count/hm^2^)	Density of lighting facilities (count/hm^2^)
Huanhuaxi Park	32.30	Comprehensive	5–7	87.70	5091	0.12	7.83
People’s Park	11.30	Comprehensive	4–5	85.50	3965	0.37	7.85
Dongpo Park	4.10	Community park	3	67.60	1802	0.24	14.88
Xiyuan Street Sports Park	6.90	Specialized park	4	59.80	10,556	0.12	16.79
Wanxiang Park	2.60	Street park	3	47.90	5776	0.27	23.24

## Research design

A study questionnaire was designed to explore resident satisfaction with the built environment of urban parks. The questionnaire had three parts. The first part surveyed the social attributes of the residents: gender, age, education level, and chronic disease. The second part surveyed the physical activity of the residents at the parks: duration, frequency, and physical activity intensity during weekly visits to the parks. The third part assessed resident satisfaction with the park’s built environment. Satisfaction with the different factors was measured using a Likert 5-point scale, with each item ranked from 1 to 5 as follows: very dissatisfied, dissatisfied, neutral, satisfied, and very satisfied.

Based on previous research, the characteristics of the built environment were sorted into 5 dimensions and 16 factors. The 5 dimensions were pathway design, park maintenance, park landscape, park facilities, and activity sites. Previous research has found these dimensions to be significantly associated with park-based physical activity. For example, Kaczynski found that trails had the strongest relationship with the use of parks for physical activity^9^, while Zhai and Baran found that park pathways with a width of between 3 and 3.9 m were heavily used by seniors^30^. Correspondingly, this study considered two factors: pathway length and width.

Another study found that park upkeep was the most important characteristic for park visitation and physical activity^13.^ Thus, this study also considered four factors relating to park maintenance: sanitation, plants, water quality, and pathway maintenance. Other studies have found that park aesthetics^31^, park facilities^14^, and activity sites^32^ were significantly associated with physical activity. In this study, park landscape included two factors: water body landscape and plant landscape. Park facilities included five factors: rest facilities, public toilets, fitness facilities, lighting facilities, and the playground. For the purpose of this study, a playground is defined as a facility designed for children to conduct physical activities. Finally, activity sites included three factors: site size, site comfort, and site facilities, such as spaces for square dancing, Tai Chi, basketball courts, volleyball courts, and other activities. The survey team also investigated environmental characteristics, such as the park area, park type, and main road width.

### Data collection

The questionnaire survey team consisted of five members, each with a professional background in landscape architecture and landscape gardening. Team members were trained to ensure they understood and were proficient with the questionnaire. The questionnaire survey was administered from April to June 2022. Each park was visited on two days: a weekday and a weekend day. Based on each respondent’s age and assessed education level, the team used either the self-administered questionnaire or the interview questionnaire. The questionnaires were both distributed and collected in the parks to ensure that the respondents were physically active in those parks. A total of 600 questionnaires were distributed, and 568 questionnaires were collected, for a recovery rate of 94.6%. Among the returned questionnaires, 514 were valid, yielding a 90.5% validity rate. The environmental survey of the five parks was conducted simultaneously.

### Methods

Descriptive statistical analyses, a chi-square test, a participant satisfaction assessment, and an ordered logistic regression analysis were performed using the statistical software package (SPSS) 27.0.

#### Participant satisfaction with the park’s built environment

The participant’s satisfaction with each environment factor was assessed along a 5-point Likert scale: 1 for very dissatisfied, 5 for very satisfied. The satisfaction value of each factor was calculated using the following equation:

1 $$\text{S}=\frac{a+2b+3c+4d+5e}{n}$$

where S is the satisfaction value of each factor, a is the very dissatisfied sample size, b is the dissatisfied sample size, c is the neutral sample size, d is the satisfied sample size, e is the very satisfied sample size, and n is the total sample size.

#### Ordered logistic regression analysis

All study variables were categorical. The dependent variables—duration and frequency—are ordered variables, so an ordered logistic regression analysis was conducted. The dependent variable duration was assigned a value as follows: 1 = less than 1 h, 2 = 1–2 h, 3 = 2–3 h, and 4 = 3 and more than 3 h. Frequency per week was assigned a value as follows: 1 = less than 1, 2 = 1–2 times, 3 = 3–6 times, and 4 = every day. The demographic (social) attributes were the control variables. Gender was assigned 1 = male or 2 = female; age was assigned 1 =  ≤ 17 years old, 2 = 18–45 years old, 3 = 46–69 years old, or 4 =  ≥ 70 years old. Completed education level was assigned 1 = Primary and below, 2 = Junior, 3 = High School (vocational school), 4 = University (College), or 5 = Postgraduate. Finally, chronic diseases were assigned as follows: 1 = none, 2 = 1–2 kinds, 3 = 3 kinds or more. The participant’s satisfaction with the built environment was the independent variable.

All methods were carried out in accordance with the Declaration of Helsinki and that all experimental protocols were approved by Ethics Committee of Sichuan Agricultural University. Informed consent was obtained from all subjects and/or their legal guardian(s).

## Results

### Reliability and validity

An assessment of the questionnaire’s validity and reliability found that the Cronbach’s α coefficient was 0.860 and the data reliability coefficient value exceeded 0.6, indicating acceptable data reliability. The KMO value was 0.855, and the Bartlett test result yielded a significance level of *p* < 0.05, indicating a good validity level.

### Descriptive statistics

Table 2 shows that of the 514 participants, 55.7 percent were male and 44.3 percent were female. Most study participants visiting the park for physical activity were young, ranging from 18 to 45 years old; middle-aged, ranging from 46 to 69 years old; followed elderly, defined as age 70 and above. Participants 17 years old or below were least likely to visit the park for physical activity. The education level for the highest proportion of participants was university graduate, with a smaller proportion of postgraduate students. Most surveyed residents were free of chronic diseases, with a few having 1–2 chronic diseases.

**Table 2:** Study participants’ demographic characteristics.

Demographic indicator	Categories	Percentage (%)
Gender	Male	55.7
	Female	44.3
Age	17 years and below	2.1
	18–45 years old	41.4
	46–69 years old	40.3
	70 years old and above	16.1
Completed education level	Primary and below	14.0
	Junior high school	23.7
	High School (vocational school)	20.4
	University (college)	35.8
	Postgraduate	6.0
Chronic diseases	None	79.6
	1–2 kinds	17.9
	3 kinds or more	2.5

**Fig. 3:**
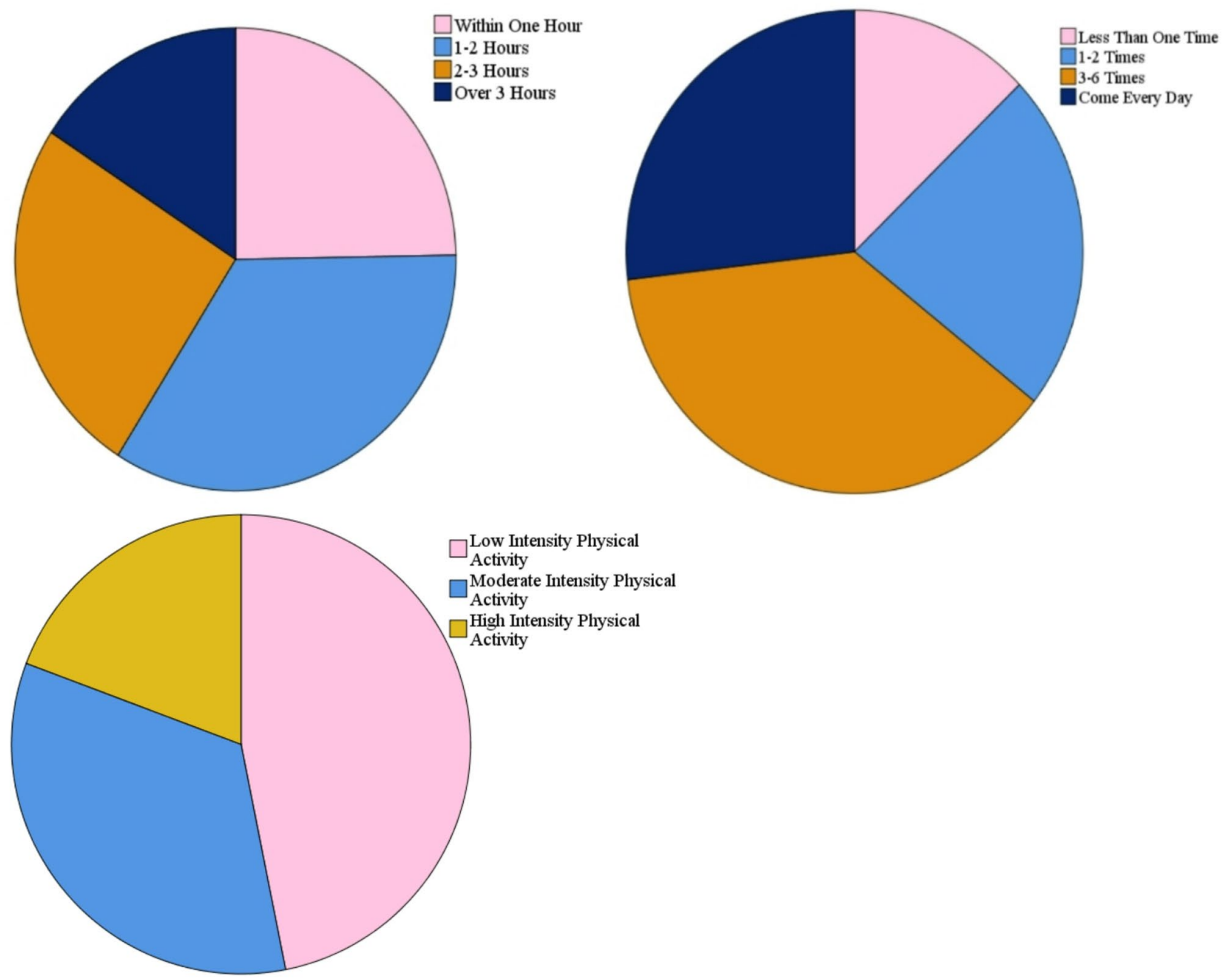
Physical activity characteristics: Duration, frequency, and intensity.

In terms of physical activity, the proportion of participants engaging in activities lasting for 1–2 h was the largest, at 34.2%, followed by participants engaging for 2–3 h, at 25.3%. The smallest proportion of participants reported staying in the park over 3 h. This result indicates that clear opportunities exist for extending the duration of park activities. The proportion of participants reporting going to the park 3–6 times a week was largest, at 35.6%; 12.84% of participants reported going less than once per week. This result indicates that opportunities also exist for residents to increase their weekly frequency of park visits. In terms of the intensity of physical activity, nearly 50% of the participants reported engaging in low-intensity physical activities, such as sitting, walking, singing, and playing music. Less than 20% of the participants reported engaging in high-intensity exercise, indicating a low level of intensity with respect to physical activity (Fig. 3).

### Correlation between social attributes and physical activity

Table 3 shows that participants’ demographic (or social) attributes significantly impacted physical activity. According to the chi-square test, gender was not significantly correlated with the duration and frequency of activity; it was, however, highly significantly correlated with intensity. Age, education level, and chronic disease were all significantly correlated with the duration, frequency, and intensity of physical activity. A significantly higher proportion of males engaged in high intensity physical activity compared to females. The 46–69-year-old age group had a higher proportion of people engaging in physical activity for 2–3 h, engaging in moderate-intensity physical activity, and visiting the park more frequently. The proportion of residents with an educational level of junior high school and below reported more activity hours, more frequent visits to the park, and more low intensity activity compared to the other groups. Participants with chronic diseases were more likely to be physically active for a longer duration, visited the park at a higher frequency, and engaged in a lower intensity of physical activity.

**Table 3:** Correlation between participant attributes and physical activity (chi-square test).

Social attributes		Duration	Frequency	Intensity
Gender	Pearson’s chi-square	1.130	1.727	13.934**
	Significant (*p* value)	0.770	0.632	0.001
Age	Pearson’s chi-square	90.080**	143.00**	65.171**
	Significant (*p* value)	0.000	0.000	0.000
Education level	Pearson’s chi-square	52.561**	68.139**	56.869**
	Significant (*p* value)	0.000	0.000	0.000
Chronic diseases	Pearson’s chi-square	34.335**	32.710**	20.423**
	Significant (*p* value)	0.000	0.000	0.000

** Indicates statistically significant at the 0.01 level.

### Participant satisfaction with built environment

As shown in Table 4, participants were highly satisfied with the plant landscape and the length and width of the pathway; they were most satisfied with the plant landscape, at a 4.139 level. Survey results indicated that the five parks have rich plant landscapes, diverse plant species, and seasonal distinctions, leading to the high level of satisfaction. However, participants were less satisfied with the facilities, especially with respect to the site facilities and playground, at 3.613 and 3.580, respectively. Finally, participants expressed the lowest satisfaction with water quality management, at 3.560.

**Table 4:** Participant satisfaction with built environment factors.

Factor	Satisfaction	Factor	Satisfaction
Plant landscape	4.139	Lighting facilities	3.814
Pathway width	4.118	Water body landscape	3.755
Pathway length	4.095	Site size	3.727
Pathway maintenance	4.048	Toilet facilities	3.691
Plant maintenance	4.025	Fitting facilities	3.640
Sanitation maintenance	4.019	Site facilities	3.613
Rest facilities	3.840	Playground	3.580
Site comfort	3.833	Water management	3.560

### Ordered logistic regression analysis of built environment on physical activity

As shown in Table 5, in addition to the significant effects of age and education, six factors affected activity duration at a significant or highly significant level: sanitation maintenance, plant landscape, site facilities, lighting facilities, public toilet facilities, and playground. Participants who were neutral with respect to sanitation maintenance tended to engage in activity for less than 1 h, compared to those who were very satisfied, who engaged for longer. Those dissatisfied with the playground also tended to engage in activity for less than 1 h; those who were neutral tended to engage in activity for 1–2 h. This result indicates that a lower satisfaction level was associated with a shorter duration of physical activity in the park. As satisfaction increased, residents extended the duration of their activity. Those who were neutral with respect to site facilities, toilet facilities, and lighting facilities tended to engage in activity for longer periods of time compared to those who were very satisfied. This result may indicate a high level of tolerance with respect to these facilities; however, it may also indicate an interest in enhancing the facilities.

**Table 5:** Ordered logistic regression model of satisfaction and the duration of physical activity.

	One hour or less		1–2 h		2–3 h	
	Odds Ratio	*p* value	Odds Ratio	*p* value	Odds Ratio	*p* value
Intercept		0.134		0.315		0.103
Gender	1.312	0.495	0.965	0.92	1.266	0.521
Age	39.704	0.000**	2.928	0.103	2.936	0.121
Education level	8.012	0.018*	5.996	0.035*	3.005	0.192
Chronic diseases	1.02	0.987	3.75	0.182	9.555	0.082
Pathway width	0.415	0.519	0.713	0.77	2.36	0.477
Pathway length	1.369	0.733	2.404	0.262	0.336	0.139
Sanitation maintenance	24.986	0.012*	11.605	0.041*	10.989	0.057
Plant maintenance	0.924	0.897	1.099	0.853	0.483	0.162
Water management	0.688	0.559	0.538	0.252	1.213	0.732
Pathway maintenance	1.569	0.493	1.276	0.657	1.444	0.514
Water body landscape	1.414	0.633	2.787	0.114	0.797	0.742
Plant landscape	3.485	0.023*	2.905	0.023*	1.751	0.249
Site size	1.45	0.600	0.656	0.484	0.695	0.572
Site facilities	0.156	0.034*	0.207	0.048*	1.351	0.718
Site comfort	1.61	0.829	0.267	0.509	1.57	0.824
Rest facilities	2.074	0.338	0.82	0.754	1.208	0.783
Toilet facilities	0.113	0.062	0.307	0.238	0.022	0.003**
Light facilities	0.058	0.002**	0.29	0.134	0.301	0.141
Fitting facilities	1.306	0.797	2.455	0.345	1.443	0.705
Playground	19.737	0.014*	7.941	0.063	3.332	0.32

* Indicates statistically significant at the 0.05 level; ** Indicates statistically significant at the 0.01 level.

The logistic regression model had a significance level of *p* < 0.05, indicating a good model fit. As shown in Table 6, six factors (in addition to age) affected the frequency of physical activity at a significant or highly significant level: pathway width, pathway length, site facilities, playground, water maintenance, and pathway maintenance. Those who were very dissatisfied with the playground tended to visit the park less than once a week; those who were neutral with respect to pathway maintenance also tended to visit the park at this rate. Those who were satisfied with the pathway width tended to visit the park 3–6 times a week. Similar to activity duration, site facilities and activity frequency were negatively correlated. The results indicate that low satisfaction with the built environment led to a lower reported visit frequency. Increasing the frequency of park visits may require increasing satisfaction levels with these factors of the built environment.

**Table 6:** Ordered logistic regression model of park satisfaction and frequency of physical activity.

	Less than 1 time		1–2 times		3–6 times	
	Odds ratio	*p* value	Odds ratio	*p* value	Odds Ratio	*p* value
Intercept		0.852		0.989		0.833
Gender	0.51	0.109	0.582	0.119	0.933	0.810
Age	192.683	0.001 **	12.868	0.075	7.574	0.140
Education level	1.290	0.835	0.590	0.590	0.591	0.542
Chronic diseases	0.110	0.241	11.850	0.988	0.602	0.536
Pathway width	0.461	0.431	0.958	0.955	4.973	0.013 *
Pathway length	1.395	0.750	0.843	0.828	0.235	0.024 *
Sanitation maintenance	1.095	0.891	1.015	0.978	0.919	0.844
Plant maintenance	1.676	0.493	0.659	0.449	0.442	0.072
water management	0.282	0.203	0.171	0.033 *	0.258	0.035 *
Pathway maintenance	8.885	0.049 *	5.330	0.070	2.377	0.286
Water body landscape	1.793	0.467	3.108	0.079	1.657	0.340
Plant landscape	0.265	0.203	0.245	0.098	0.567	0.406
Site size	0.629	0.748	4.902	0.076	2.046	0.361
Site facilities	0.115	0.021 *	0.525	0.418	1.080	0.910
Site comfort	4.857	0.461	1.268	0.890	0.327	0.437
Rest facilities	0.764	0.720	1.224	0.747	1.674	0.332
Toilet facilities	1.935	0.477	0.397	0.180	1.235	0.733
Lighting facilities	2.768	0.244	1.273	0.716	1.264	0.673
Fitting facilities	0.756	0.778	0.723	0.699	0.453	0.284
Playground	4.716	0.996	49.443	0.036 *	1.647	0.993

* Indicates statistically significant at the 0.05 level; ** Indicates statistically significant at the 0.01 level.

## Discussion

### Analysis of how built environment factors affect physical activity

This study found that site facilities and a playground affected the duration and frequency of physical activity, an outcome consistent with previous research^13,33^. In a study by Van Hecke, the presence of a playground and sport fields was important to adolescents when visiting parks and engaging in physical activity^13^. In a separate study, entertainment facilities and sports equipment were found to be significantly correlated to resident activity^33^. In this study, participants reported low satisfaction with both of these factors. One explanation may be that People’s Park and Dongpo Park lack sports fields such as basketball courts. Another could be that the children’s playground in People’s Park requires an entry fee.

This study also found that public toilet and lighting facilities were highly significantly correlated with the duration of physical activity. The finding is consistent with other studies. A similar study in Hong Kong found that the quality of park amenities was positively related to children’s physical activity in parks^34^. Furthermore, public access to toilets and lighting around courts have been associated with increased park use by adolescents^35^. The availability of public toilet facilities is important for people engaging in physical activities, and the number and quality of the public toilets also reflect the quality of the park. Lighting facilities, in particular, relate to park safety; lighting at night increases safety and illuminates the beauty of the park.

This study found that the three factors of sanitation maintenance, pathway, and water maintenance were significantly correlated with the duration and frequency of physical activity. These results are consistent with previous research, which found that park upkeep was the factor that most affected visitation and physical activity^13^. For example, the water features in the parks of Aydin City, Turkey, were found to be negatively correlated with the frequency of physical activity due to water pollution^36^. In this study, participants expressed a low level of satisfaction with water management, which is an important factor to consider.

Previous studies have observed that high-quality and accessible walking paths are a preferred park feature for older adults^15^. One study found that the number of park visitors, regardless of age, increased after paved walking and cycling paths were installed^37^. Another study found that participants appreciated a path wide enough to allow space for walking in both directions, as multiple roads increase walking options^31^. Similarly, this study found that pathway length and width were significantly associated with a higher frequency of physical activity. People who were satisfied with the path width and length visited the park at the higher frequency, 3–6 times a week. The main road width in Huanhuaxi Park is 5–7 m, reflected in the highest level of satisfaction reported by participants. The proportion of residents visiting the park 3–6 times a week was higher in Huanhuaxi Park than Dongpo Park, which has a main road width of 3 m.

The plant landscape has a positive and significant relationship with the physical activity levels of children^3^. Specifically, a strong relationship exists between the richness of the natural scenery and the activity level of children^38^. De Vries et al. found that high vegetation cover was associated with a high level of physical activity^39^. A larger green space, achieved through park greening in urban parks, was also associated with a higher frequency of park visitation^40^.

In this study, plant landscapes were significantly correlated with the duration of physical activity: a higher level of satisfaction with the plant landscape was associated with an increased duration of activity. Participant satisfaction with the green coverage in Huanhuaxi Park was the highest, at 4.426. The percentage of people who reported being active for more than 2–3 h in this park was higher than in Dongpo Park or Xiyuan Street Sports Park, both of which have low green coverage.

### Cause analysis of how built environment factors impact physical activity

The research above identified ten factors of the built environment that influence the duration and frequency of physical activity: site facilities, playground, light facilities, toilet facilities, sanitation maintenance, water maintenance, pathway maintenance, pathway width, pathway length, and plant landscape. These ten factors were sorted along five dimensions: activity site, park facilities, park maintenance, pathway design, and park landscape. The activity site includes the site facilities. The park facilities include light facilities, toilet facilities, and playground. Park maintenance includes sanitation, water, and pathway maintenance. Pathway design includes pathway length and width. Finally, park landscape includes the plant landscape. An in-depth cause analysis of these factors may lead to a better understanding of their roles.

For example, an activity site impacts physical activity because of the space and related opportunities for engaging in physical activity. Providing a range of options to cater to different age groups, interests, and abilities can enhance the appeal and functionality of parks. Recreational facilities increase the interactivity and fun of parks, encouraging repeat visits^34^.

Park facilities impact physical activity because they provide facilitation and support for engaging in those types of activities^35^, increasing the willingness of residents to visit parks and lengthening the amount of time they spend there. Park facilities are important for effectively engaging in physical activity^41,42^; thus, the availability of park facilities encourage people to engage in physical activity^9^.

Park maintenance impacts physical activity because it impacts residents’ perceptions of the environment. Specifically, clean and tidy sanitation facilities increase residents’ positive perceptions of the park aesthetics^43^. A neat and smooth path improves perceptions of safety, and clear and clean water improves perceptions of comfort. Park maintenance fosters a sense of pride and ownership among park users^44^, encouraging them to spend time in the park and engage in physical activities.

Pathway design impacts physical activity because different designs can support a variety of activities and improve residents’ comfort when engaging in those activities. Pathways of appropriate length and width can facilitate multiple activities that appeal to people of different ages and skill levels^9^, such as walking, cycling, and rollerblading. Pathways paved with soft, safe materials such as plastic and brick can help cushion the pressure of walking^30^, improving physical comfort.

The park landscape impacts physical activity because it promotes mental and physical well-being through its aesthetics. Trees, plants, and green spaces act as natural air filters, removing harmful substances, releasing oxygen, and providing cleaner air for overall well-being ^45^. The plant landscape, an important part of the natural landscape, has been linked to a notable decrease in stress levels^46^. The presence of trees, bushes, gardens, grass, flowers, and water features contributes to park aesthetics. Fresh air and the smells of nature enhance park aesthetics and increase the duration of park visits ^47^.

### Proposed design strategy to promote physical activity in Chengdu urban parks

This study explored how the built environments of urban parks impact residents’ physical activities in Chengdu. Based on the results, several design strategies are proposed that may further promote physical activity.

Many studies have shown that the quantity and quality of park sites and facilities can promote physical activity^34,44^. In Chengdu, many of the urban parks lack dedicated sports areas, such as basketball courts and table tennis tables. There are often not enough playgrounds for children. This study highlights the need for increasing the number and type of activity sites and facilities. In terms of playgrounds, in addition to conventional slides, priority should be given to physically challenging play equipment^48^, such as climbing pools, swings, and extreme bikes. This type of equipment would better stimulate children’s desires for challenges and promote physical activity. One effective way to improve facility quality is to provide well-maintained facilities, such as drinking fountains, toilets, picnic tables, and lighting^34^. These facilities support physical activity over a long period of time^35^ as well as increase safety^34^. In addition, integrating intelligent and technological elements increases the interaction between the facilities and residents, attracting more people to participate in physical activities.

This study found that park maintenance and pathway design were closely related to the duration and frequency of physical activity. Measures for improving park maintenance include engaging in intensive and regular park maintenance^13^, ensuring that the ground is clean and tidy, providing sufficient rubbish bins across the park, ensuring that road surfaces are smooth and free of potholes, and using non-slip and comfortable paving material. Park water should be clean and odorless, with regular salvage and clean-up operations to remove rubbish and leaves. Fish, ducks, and other animals could also be introduced to increase the aesthetics of the park. Chengdu is rich in water resources, and many city parks have abundant water bodies such as lakes, pools, and rivers. Good water maintenance could increase park attractiveness. With respect to road design, park design specifications indicate that the main road width in a comprehensive urban park should be 4–5 m and the width in a community park should be 2.5–4.5 m^49^. Different materials and colors would help differentiate pathway areas (i.e., cycling paths versus walking paths) and prevent interference between the various activities. For example, a study by Paudel found that older adults prefer rubber surfaces ^50^. The appropriate design of walking loops, with branches to other areas, provides a variety of options^51^.

De Vries et al. found that high vegetation cover was associated with a high level of physical activity^39^. Thus, the vegetation cover of green areas needs to be increased. The introduction of shade-providing tree species and vertical greening should be prioritized^36^. Finally, an attractive plant landscape can be created by planting trees, shrubs, and grasses to maintain plant diversity^52^, and matching plant colors and seasonal effects to increase attraction.

## Conclusions

This empirical study explored the factors of a built environment that affect residents’ physical activities in urban parks in Chengdu, China. The main findings and contributions of the study are as follows. Sanitation maintenance, plant landscape, playground, site facilities, lighting facilities, and public toilet facilities significantly affected the duration of physical activity. The frequency of physical activity was significantly impacted by pathway maintenance, playground, pathway width, pathway length, site facilities, and water quality management. The study also analyzed causes of how the built environment of parks influences physical activity. The results suggest design optimization strategies for Chengdu urban parks in particular, and can serve as a reference for other cities and countries.

### Limitations and prospects

This research focused on built environment factors, which are extensively related to physical activity. However, there may be additional factors that were not addressed. Future research should focus on specific aspects of the built environment in urban parks, such as park trails and vegetative greenness. This research also found that the social attributes of individuals, such as age and education level, significantly affected the duration and frequency of their physical activity. Further studies are needed to identify the effect of factors impacting specific populations, so urban parks can be designed to meet their needs. Finally, in addition to the elderly and children, more attention should be paid to the middle-aged and youth populations.

## Data Availability

The data used and/or analyzed during the current study available from the corresponding author on reasonable request.
